# *GNB3* c.825c>T polymorphism influences T-cell but not antibody response following vaccination with the mRNA-1273 vaccine

**DOI:** 10.3389/fgene.2022.932043

**Published:** 2022-08-29

**Authors:** Ieva Čiučiulkaitė, Birte Möhlendick, Laura Thümmler, Neslinur Fisenkci, Carina Elsner, Ulf Dittmer, Winfried Siffert, Monika Lindemann

**Affiliations:** ^1^ Institute of Pharmacogenetics, University Hospital Essen, University of Duisburg-Essen, Essen, Germany; ^2^ Institute for Transfusion Medicine, University Hospital Essen, University of Duisburg-Essen, Essen, Germany; ^3^ Institute for Virology, University Hospital Essen, University of Duisburg-Essen, Essen, Germany

**Keywords:** *GNB3* c.825C>T, COVID-19, SARS-CoV-2, mRNA-1273, antigen-specific T-cell response, ELISpot, SARS-CoV-2 spike antibody titer

## Abstract

**Background:** Immune responses following vaccination against COVID-19 with different vaccines and the waning of immunity vary within the population. Genetic host factors are likely to contribute to this variability. However, to the best of our knowledge, no study on G protein polymorphisms and vaccination responses against COVID-19 has been published so far.

**Methods:** Antibodies against the SARS-CoV-2 spike protein and T-cell responses against a peptide pool of SARS-CoV-2 S1 proteins were measured 1 and 6 months after the second vaccination with mRNA-1273 in the main study group of 204 participants. Additionally, antibodies against the SARS-CoV-2 spike protein were measured in a group of 597 participants 1 month after the second vaccination with mRNA-1273. Genotypes of *GNB3* c.825C>T were determined in all participants.

**Results:** The median antibody titer against the SARS-CoV-2 spike protein and median values of spots increment in the SARS-CoV-2 IFN-γ ELISpot assay against the S1-peptide pool were significantly decreased from months 1 to 6 (*p* < 0.0001). Genotypes of *GNB3* c.825C>T had no influence on the humoral immune response. At month 1, CC genotype carriers had significantly increased T-cell responses compared to CT (*p* = 0.005) or TT (*p* = 0.02) genotypes. CC genotype carriers had an almost 6-fold increased probability compared to TT genotype carriers and an almost 3-fold increased probability compared to T-allele carriers to mount a SARS-CoV-2-specific T-cell response above the median value.

**Conclusion:** CC genotype carriers of the *GNB3* c.825C>T polymorphism have an increased T-cell immune response to SARS-CoV-2, which may indicate better T-cell-mediated protection against COVID-19 after vaccination with mRNA-1273.

## 1 Introduction

Antibodies and T-cells play an important role in both the outcome of COVID-19 and vaccination against it. Interaction between the angiotensin-converting enzyme 2 receptor expressed on the host cells and the receptor-binding domain in the spike (S) 1 subunit of the SARS-CoV-2 spike protein allows the virus to enter the host cell (Harrison et al., 2020). Vaccines against COVID-19 encode this SARS-CoV-2 spike protein and induce an immune response (Martinez-Flores et al., 2021). Immune responses following vaccination against COVID-19 with different vaccines and the waning of immunity vary within the population (Collier et al., 2021). Common factors such as age, sex, pre-existing conditions, or immunosuppressive therapy have been investigated and shown to contribute to this variability (Geisen et al., 2021; Lindemann et al., 2021; Simon et al., 2021; Steensels et al., 2021; Widge et al., 2021). In addition, genetic host factors are also likely to contribute to this variability (Crocchiolo et al., 2022; Gutierrez-Bautista et al., 2022). However, to the best of our knowledge, no studies on vaccination against COVID-19 and G protein polymorphisms have been published so far.

Here, we investigated whether genotypes of the c.825C>T polymorphism in the gene *GNB3* (rs5443) may influence the immune response after vaccination against COVID-19. This polymorphism exerts diverse influences on G protein-mediated signaling by generating a splice variant of the G protein subunit beta-3 (Siffert et al., 1998). Previous studies have shown that the *GNB3* c.825C>T polymorphism affects the immune response after stimulation with various recall antigens and after vaccination against the hepatitis B virus (HBV) (Lindemann et al., 2001; Lindemann et al., 2002).

## 2 Methods

### 2.1 Study group

For the study, 2,526 healthcare workers from the University Hospital Essen (Essen, Germany) were recruited. From this study cohort, we gathered a homogeneous group of 204 participants aged between 18–40 years for further investigations. All participants in this study group were non-obese, non-smokers, and were healthy or had minor health issues, but no immunosuppressive conditions or cardiovascular diseases. Immune responses after the vaccination in the study group did correlate neither with age nor with BMI. Furthermore, there were no differences in immune responses between healthy participants and participants with minor health issues. The allele frequencies of *GNB3* c.825C>T are differently distributed in African and East Asian populations. In this study, merely two participants belonged to these populations and they constituted less than 1% of our study group. The selection was based on questionnaires and the flow chart of enrollment is shown in Supplementary Figure S1. For additional investigations of antibody titers, we established an age-matched replication group of 597 participants. All participants in both study groups were vaccinated twice with the COVID-19 vaccine mRNA-1273 (Moderna Inc.). None of the participants had a history of SARS-CoV-2 infection and all tested negative for antibodies against the SARS-CoV-2 nucleocapsid protein. The investigations were reviewed and approved by the Ethics Committee of the Medical Faculty of the University of Duisburg-Essen (21–10005-BO). All participants provided their written informed consent to participate in this study.

### 2.2 Study design

Blood samples were taken from all participants 1 and 6 months after the second vaccination with mRNA-1273. We measured antibody titers against the SARS-CoV-2 S protein and the SARS-CoV-2 nucleocapsid protein and determined genotypes of the *GNB3* c.825C>T polymorphism. In addition, in the main study group of 204 participants, the T-cell response against the S1 peptide pool was measured using the SARS-CoV-2 IFN-γ ELISpot assay 1 and 6 months after the second vaccination.

### 2.3 *GNB3* c.825C>T genotyping

Genomic DNA was extracted from 200 µl EDTA blood using the QIAamp® DNA Blood Mini Kit (Qiagen, Hilden, Germany). Polymerase chain reaction (PCR) was performed with 2 µl genomic DNA and 30 µl Taq DNA-Polymerase 2x Master Mix Red (Ampliqon, Odense, Denmark) under the following conditions: initial denaturation 94°C for 3 min, 38 cycles with denaturation at 94°C for 30 s, annealing at 60°C for 30 s, elongation at 72°C for 30 s each, and final elongation at 72°C for 10 min (forward primer: 5′ GCC​CTC​AGT​TCT​TCC​CCA​AT 3'; reverse primer 3′ CCC​ACA​CGC​TCA​GAC​TTC​AT 5′). PCR products were digested with BseDI (Thermo Scientific, Dreireich, Germany), and restriction fragments were analyzed by agarose gel electrophoresis. For the various genotypes, results from restriction fragment length polymorphism (RFLP)-PCR were validated by Sanger sequencing.

### 2.4 Detection of antibodies against SARS-CoV-2 spike protein

Determination of anti-spike SARS-CoV-2 antibody concentrations was performed using the SARS-CoV-2 S1 receptor-binding domain (RBD) IgG/sCOVG test (Siemens Healthcare GmbH, Erlangen, Germany) according to the manufacturer’s instructions. Anti-Spike SARS-CoV-2 antibody concentration results were reported in binding antibody units per ml (BAU/ml). The limit of detection for positivity was 21.8 BAU/ml.

### 2.5 Detection of antibodies against the SARS-CoV-2 nucleocapsid protein

All samples were also analyzed for SARS-CoV-2 IgG antibodies against the nucleocapsid protein to exclude participants with prior SARS-CoV-2 infection. The Architect i2000SR CoV-2 IgG assay (Abbott Diagnostics, IL, United States) was used according to the manufacturer’s instructions. Results with an index ≥1.4 were considered evidence of the previous infection.

### 2.6 ELISpot assay

To assess SARS-CoV-2-specific cellular immunity, we performed ELISpot assays using an overlapping peptide pool of SARS-CoV-2 S1 proteins (Miltenyi Biotec, Bergisch Gladbach, Germany) without the addition of any cytokines. We tested 250,000 peripheral blood mononuclear cells (PBMCs) per sample and measured IFN-γ production after 20 h of incubation. Mean values of duplicate cell cultures were considered. The median and mean spot numbers of autologous (unstimulated) controls were 0 and 0.05, respectively. SARS-CoV-2-specific spots were determined as stimulated minus unstimulated values (spots increment). The cut-off definition for positive results was based on negative control values (non-stimulated cultures) and the consideration that three times higher values for stimulated versus non-stimulated cells in cellular assays are often interpreted as a positive T-cell response. Using these criteria, the cut-off was 1.5 spot increments. Further details on the ELISpot assay and the cut-off definition have been published previously (Schwarzkopf et al., 2021).

### 2.7 Statistical analysis

Statistical analysis was performed with GraphPad Prism 7 (Graph Pad Software, San Diego, California, United States) and IBM SPSS Statistics 27 (IBM Software, Ehningen, Germany). Comparisons between three groups were made using the Kruskal–Wallis test and between two groups using the Mann–Whitney test. For genetic associations, we calculated the odds ratio (OR) and 95% confidence interval (CI) by Fisher’s exact test using the Baptista–Pike method for the OR. *p*-values are given two-sided and values <0.05 were considered significant.

## 3 Results

### 3.1 Descriptive statistics of study groups

In the main study group of 204 participants, 105 participants were tested 1 month and 163 participants 6 months after the second vaccination with mRNA-1273. At both time points, sixty-four subjects participated. At month 1, the median age of the study group was 24 years (range 18–39), the BMI 22.5 kg/m^2^ (range 17.0–29.9), and 69.5% (n=73) of participants were female. At month 6, the median age was 26 years (range 18–40), the BMI was 22.5 kg/m^2^ (range 17.0–29.1), and 75.5% (n=123) were female. In the additional study group of 597 participants, the median age was 28 years (range 18–40), the BMI was 23.0 kg/m^2^ (range 16.7–53.8), and 74.4% (n=444) of the participants were female.

### 3.2 Antibody titer against SARS-CoV-2 S1-RBD and T-cell response to SARS-CoV-2 S1 ELISpot assay one and six months after the second vaccination with mRNA-1273

**FIGURE 1:**
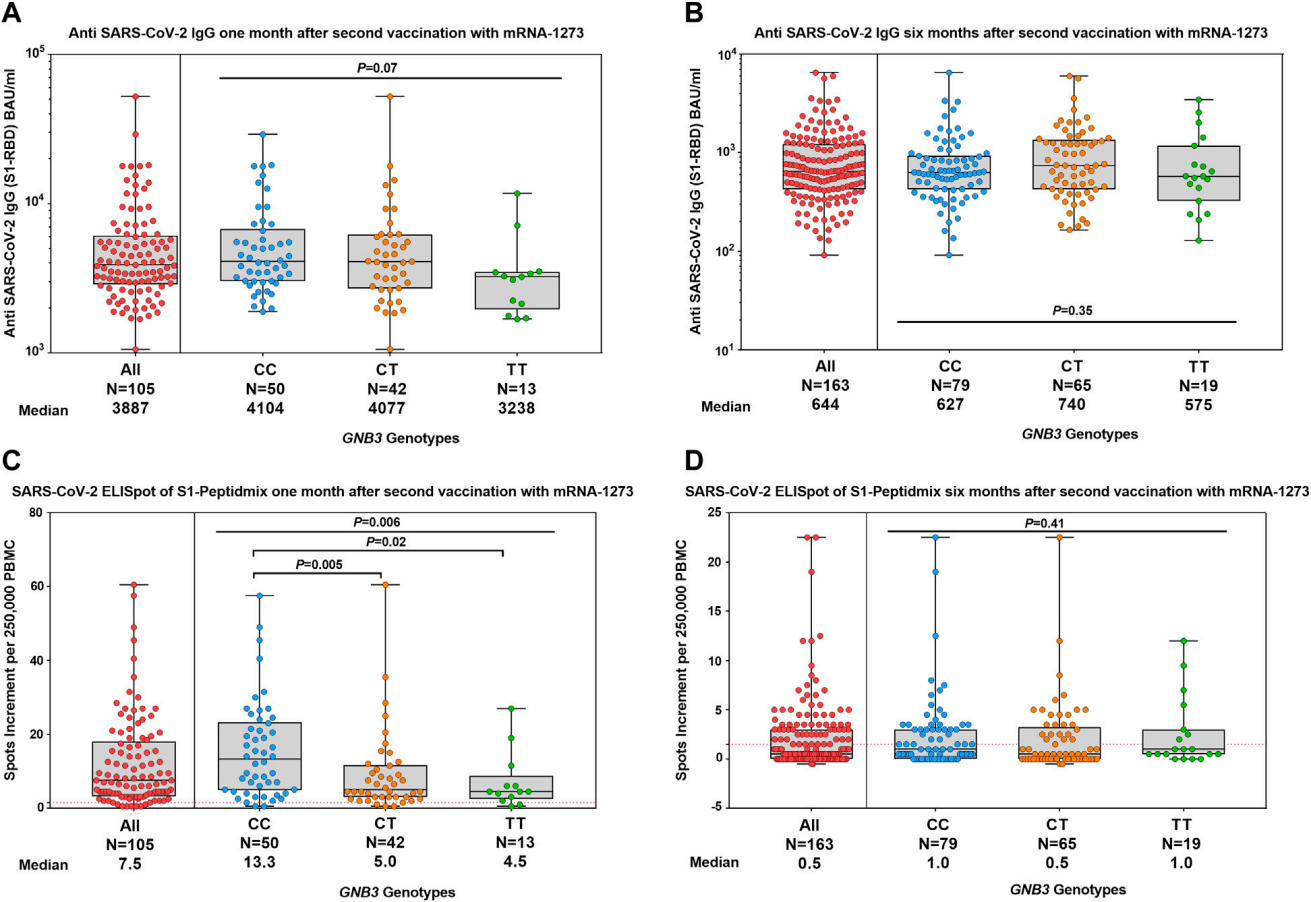
Humoral and cellular immune responses were stratified by *GNB3* genotypes 1 and 6 months after the second vaccination with mRNA-1273. Distribution of antibody concentrations against SARS-CoV-2 S1-RBD 1 month **(A)** and 6 months **(B)** after the second vaccination. ELISpot responses to the S1-protein of SARS-CoV-2 at 1 month **(C)** and 6 months **(D)** after the second vaccination. Antibody titers are reported in BAU/ml and T-cell response as spots increment. Red dashed lines indicate the cut-off for positivity (1.5 spots increment per 250,000 peripheral blood mononuclear cells).

The median antibody titer against SARS-CoV-2 S1-RBD was 3,887 BAU/ml (range 1,058–52,213) at month 1 (Figure 1A), which significantly (*p* < 0.0001) decreased to 644 BAU/ml (range 91–6,491) at month 6 (Figure 1B).

At month 1, 93.3% and at month 6, 41.7% of participants had a positive T-cell response in the SARS-CoV-2 IFN-γ ELISpot assay against the S1-peptide pool. Median values of spots increment decreased from 7.5 (range 0.5–60.5) to 0.5 (range -0.5–22.5) (*p* < 0.0001, Figures 1C,D).

**FIGURE 2:**
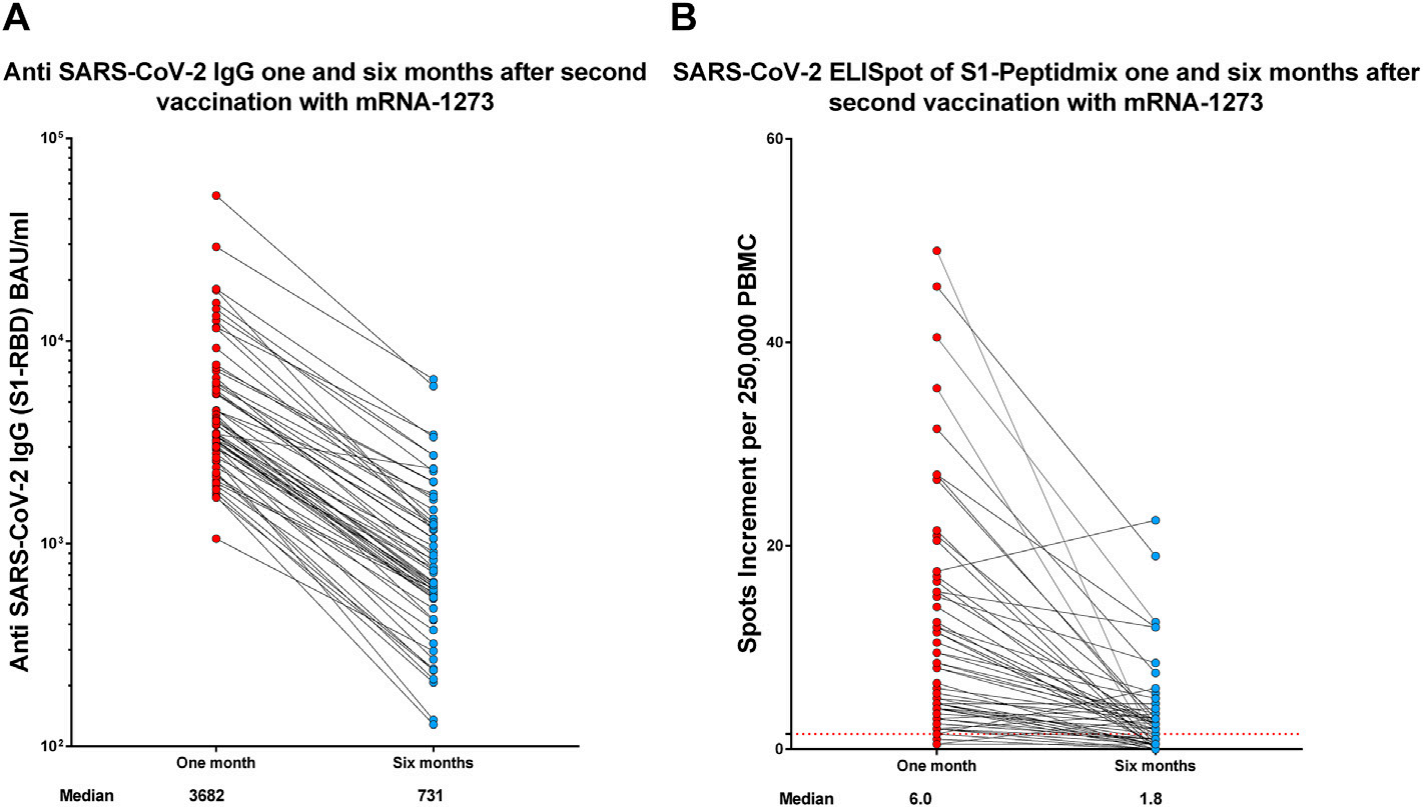
Humoral **(A)** and cellular **(B)** immune response 1 and 6 months after the second vaccination with mRNA-1273 in the group of 64 participants who were available at both time points. Antibody titers are reported in BAU/ml and T-cell response as spots increment. Red dashed lines indicate the cut-off for positivity (1.5 spots increment per 250,000 peripheral blood mononuclear cells).

Samples of 64 participants were available at both time points, 1 and 6 months after the second vaccination. The median antibody titer against SARS-CoV-2 S1-RBD was 3,682 BAU/ml (range 1,058–52,213) at month 1, which significantly (*p* < 0.0001) decreased to 731 BAU/ml (range 128–6,491) at month 6 (Figure 2A). Median values of spots increment in the SARS-CoV-2 IFN-γ ELISpot assay against the S1-peptide pool decreased from 6.0 (range 0.5–49) to 1.8 (range 0.0–22.5) (*p* < 0.0001, Figure 2B).

### 3.3 Influence of *GNB3* c.825C>T on the immune response one and six months after the second vaccination with mRNA-1273

**FIGURE 3:**
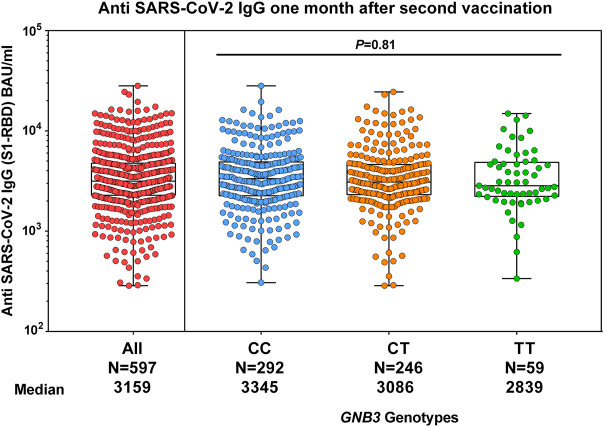
Comparison of humoral response and *GNB3* genotypes 1 month after the second vaccination with mRNA-1273 in the replication group. The median of anti-spike antibody levels is given in BAU/ml.

We investigated the impact of *GNB3* c.825C>T genotypes on the humoral immune response. We found a slightly albeit non-significantly lower anti-spike antibody titer in TT genotype carriers at month 1, which was no longer detectable in month 6 (Figures 1A,B). We validated these results in a larger cohort of 597 individuals (Figure 3).

At month 1, the median values of spots increment in the ELISpot assay were 13.3 (range 0.5–57.5) for CC, 5.0 (range 0.5–60.5) for CT, and 4.5 (range 0.5–27.0) for TT genotype carriers (*p* = 0.006, Figure 1C). CC genotype carriers had significantly increased T-cell responses compared to CT or TT genotypes (*p* = 0.005 and *p* = 0.02, respectively, Figure 1C). The effect was even more pronounced when comparing the CC genotype with T-allele carriers (13.3 vs. 4.5 spots increment, *p* = 0.001). At month 6, T-cell responses were strongly reduced and, therefore, genotype-dependent differences were no longer detectable (Figure 1D).

We analyzed the frequency distribution of *GNB3* genotypes above and below the median of 7.5 spots increment 1 month after the second vaccination to estimate if there is a genotype-related probability for a T-cell response above this cutoff. We found that CC genotype carriers had an almost 6-fold increased probability compared to TT genotype carriers (OR: 5.9, 95% CI: 1.6–21.5, *p* = 0.01) and an almost 3-fold increased probability compared to T-allele carriers (OR: 2.9, 95% CI: 1.3–6.2, *p* = 0.01) to mount a SARS-CoV-2-specific T-cell response above the median value.

## 4 Discussion

In this study, we observed a nearly 6-fold decrease in antibody titers from 1 to 6 months after the second vaccination with mRNA-1273; nevertheless, all participants remained seropositive 6 months after the second vaccination. Despite many studies on the immune response after vaccination against COVID-19, there are only a few studies on the course of antibody titers over a longer period of time after vaccination with mRNA-1273. Those studies also reported a significant drop in antibody titers after vaccination with mRNA-1273 (Collier et al., 2021; Doria-Rose et al., 2021; Tre-Hardy et al., 2021; Gallagher et al., 2022).

In addition, Tré-Hardy *et al.* investigated whether different demographic characteristics such as age, BMI, or pre-existing conditions may influence the kinetics of antibody titers and found no statistically significant relationship. Despite a very homogeneous study group of young, non-obese, and non-smoking participants without systemic immunosuppressive therapies or serious pre-existing conditions, we observed an almost 6-fold decrease in antibody titers, which confirms the findings of Tre-Hardy et al. (2021).

Some individuals generate lower antibody titers due to older age, pre-existing conditions, or immunosuppressive therapy (Geisen et al., 2021; Lindemann et al., 2021; Simon et al., 2021; Steensels et al., 2021; Widge et al., 2021). In our study, we present, for the first time, data on the potential influence of a G protein polymorphism on the immune response after vaccination with mRNA-1273. For this project, we chose the *GNB3* c.825C>T polymorphism because it was shown to correlate with T-cell responses to vaccination against HBV and to different recall antigens (Lindemann et al., 2001; Lindemann et al., 2002). In our current study, we observed that C-allele carriers had higher antibody titers, but this trend escaped statistical significance. In addition, no statistically significant differences were found between the genotypes of *GNB3* c.825C>T and the antibody titers after the booster vaccination against HBV (Lindemann et al., 2002). However, it has been shown that CT genotype carriers tend to have higher antibody titers after booster vaccination against HBV. It seems that *GNB3* c.825C > T may have a slight impact on the humoral immune response.

Data on T-cell kinetics after the vaccination with mRNA-1273 are scarce. Many studies tested T-cell immunity only once after vaccination or after a very short follow-up time. However, Gallagher *et al.* investigated the kinetics of T-cell responses after vaccination with mRNA-1273 at long-term follow-up and demonstrated approximately 30% decreased T-cell responses at a median of 223 days after the first vaccination with mRNA-1273 (Gallagher et al., 2022).

Our analysis of the cellular immunity also reveals a decrease in T-cell responses 6 months after the second vaccination with mRNA-1273. We observed a 15-fold decrease in T-cell responses in the SARS-CoV-2 ELISpot assay against the S1 peptide pool from 1 to 6 months after the second vaccination. In addition, at 6 months, only half of the participants had a T-cell response above the cut-off. It is also worth noting that our study is the first to measure T-cell responses in such a large cohort and, in addition, all tests were performed on freshly collected PBMC.

Our data show that CC genotype carriers have a stronger T-cell-mediated response and may be better protected against COVID-19 or have a milder COVID-19 infection after vaccination with mRNA-1273. This may also be an advantage for CC genotype carriers when antibodies cannot neutralize the virus and T-cell immunity is critical, e.g., after infection with immune escape variants of SARS-CoV-2 or when the humoral immune response is impaired.

However, at this time point, further studies are needed. First, our data should be replicated in an independent cohort. Further studies after booster vaccination causing a stronger immune response are also needed to see the influence of *GNB3* c.825C>T on the T-cell response after a longer follow-up. Last, the molecular mechanisms by which the *GNB3* c.825C>T polymorphism influences the T-cell response after SARS-CoV-2 vaccination and the potential clinical implications of these findings are to be yet unraveled.

## Data Availability

The original contributions presented in the study are included in the article/Supplementary Material; further inquiries can be directed to the corresponding author.
