# Supplementary material for: GNB3 c.825c>T polymorphism influences T-cell but not antibody response following vaccination with the mRNA-1273 vaccine
Source: Front Genet. 2022 Aug 29;13:932043. doi: 10.3389/fgene.2022.932043 (PMC9465595; doi:10.3389/fgene.2022.932043)

Supplementary Figure 1. Enrollment of participants for SARS-CoV-2-specific cellular immunity assessment with ELISpot assay one and six months after the second vaccination with mRNA-1273.

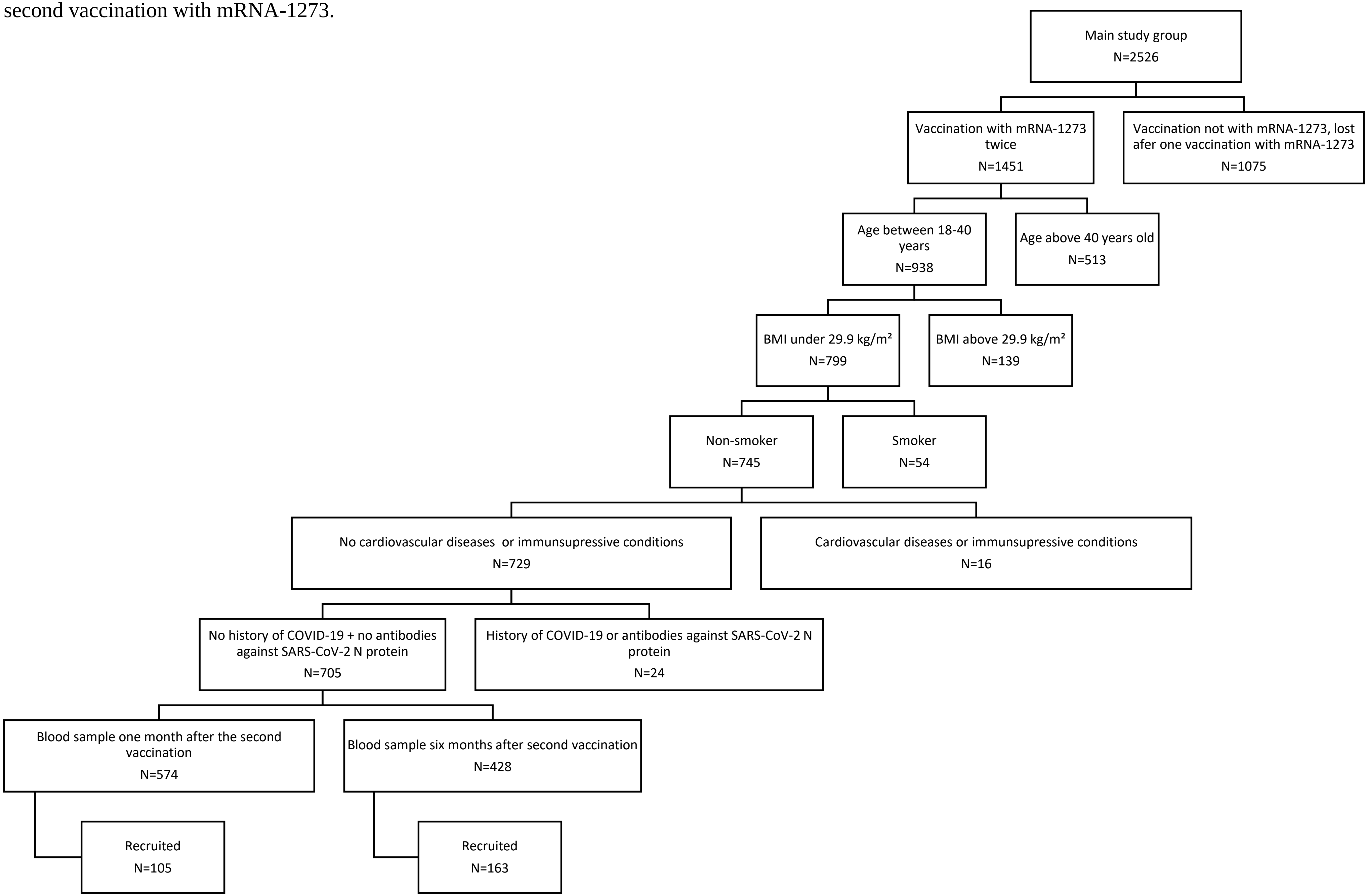

Supplement: Supplementary file 1 [file Image1.pdf]
